# Overweight as a biomarker for concomitant thyroid cancer in patients with Graves’ disease

**DOI:** 10.3389/fendo.2024.1382124

**Published:** 2024-04-22

**Authors:** Joonseon Park, Solji An, Ja Seong Bae, Jeong Soo Kim, Kwangsoon Kim

**Affiliations:** Department of Surgery, College of Medicine, The Catholic University of Korea, Seoul, Republic of Korea

**Keywords:** Graves’ disease, thyroid cancer, overweight, thyroid stimulating hormone receptor antibodies, BMI - body mass index

## Abstract

The incidence of concomitant thyroid cancer in Graves’ disease varies and Graves’ disease can make the diagnosis and management of thyroid nodules more challenging. Since the majority of Graves’ disease patients primarily received non-surgical treatment, identifying biomarkers for concomitant thyroid cancer in patients with Graves’ disease may facilitate planning the surgery. The aim of this study is to identify the biomarkers for concurrent thyroid cancer in Graves’ disease patients and evaluate the impact of being overweight on cancer risk. This retrospective cohort study analyzed 122 patients with Graves’ disease who underwent thyroid surgery at Seoul St. Mary’s Hospital (Seoul, Korea) from May 2010 to December 2022. Body mass index (BMI), preoperative thyroid function test, and thyroid stimulating hormone receptor antibody (TR-Ab) were measured. Overweight was defined as a BMI of 25 kg/m² or higher according to the World Health Organization (WHO). Most patients (88.5%) underwent total or near-total thyroidectomy. Multivariate analysis revealed that patients who were overweight had a higher risk of malignancy (Odds ratios, 3.108; 95% confidence intervals, 1.196–8.831; *p* = 0.021). Lower gland weight and lower preoperative TR-Ab were also biomarkers for malignancy in Graves’ disease. Overweight patients with Graves’ disease had a higher risk of thyroid cancer than non-overweight patients. A comprehensive assessment of overweight patients with Graves’ disease is imperative for identifying concomitant thyroid cancer.

## 1 Introduction

Graves’ disease (GD) is an autoimmune disease that causes hyperthyroidism by stimulating the thyroid gland to produce excessive thyroid hormone due to the presence of thyroid stimulating hormone receptor antibody (TR-Ab) (1–4). Surgical intervention is required for the management of GD in cases of failed medical therapy, severe or rapidly progressing disease with compressive symptoms, concomitant thyroid cancer, worsening Graves’ ophthalmopathy, or based on patient’s preference (1, 5–7).

The reported incidence of concomitant thyroid cancer in patients with GD varies, ranging from 1% to 22%, and some studies reported that the incidence of thyroid cancer is higher in patients with GD than the incidence in the general population (8–11). Although the relationship between GD and thyroid cancer is unclear, GD can make the diagnosis and management of thyroid nodules more challenging (12–16). In patients with GD and concomitant thyroid cancer, most surgeries are planned after nodules are diagnosed by ultrasound or fine-needle aspiration biopsy (FNAB). However, thyroid cancer is occasionally identified incidentally in the pathologic examination after surgery (17–19). These cases are indications that surgery was necessary, and cancer could have been missed if surgery had not been performed for other reasons. Therefore, identifying biomarkers for concomitant thyroid cancer in patients with GD may facilitate planning the surgery and more thorough screening, even if a nodule is not discovered before surgery.

Previous studies have identified risk factors for concomitant thyroid cancer in patients with GD, including TR-Ab, preoperative nodules, previous external radiation, and younger age (13, 20–24). Regardless of the existence of GD, morbid obesity affects the incidence and aggressiveness of thyroid cancer in euthyroid patients (25–29). However, few studies have investigated the relationship between thyroid cancer in patients with GD and obesity. In a study of 216 GD patients, those with thyroid cancer had significantly higher body mass index (BMI) compared to those without thyroid cancer (30). Since weight loss is common in patients with GD (31), investigations into the relationship between being overweight or obese and GD are needed. The aim of this study was to identify biomarkers for concurrent thyroid cancer in patients with GD and identify the effects of being overweight on cancer risk.

## 2 Materials and methods

### 2.1 Patients

**Figure 1:**
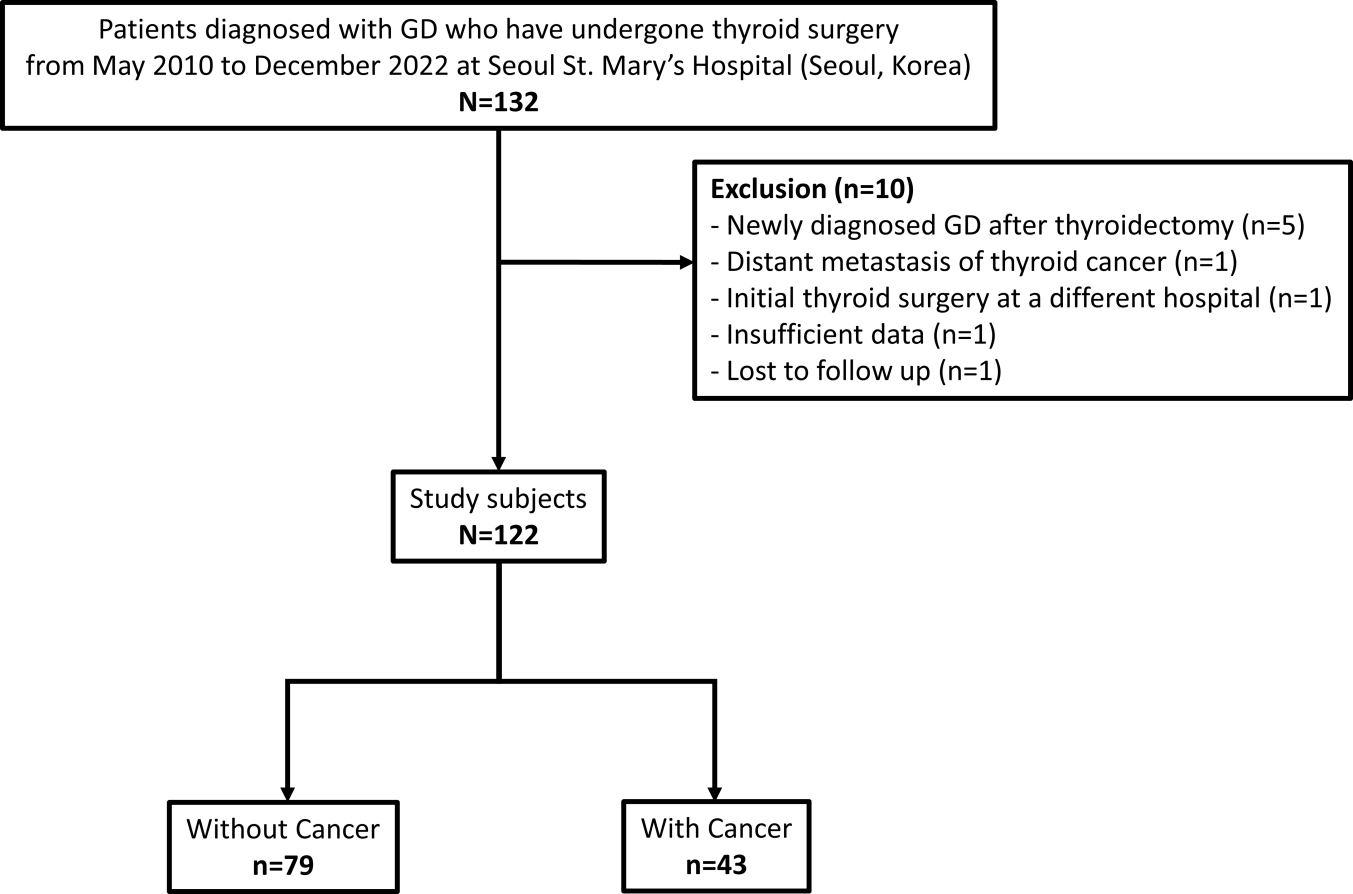
Participant flow diagram of patient selection. GD, Graves’ disease.

We retrospectively reviewed the medical charts and pathology reports of 132 patients with GD who underwent thyroid surgery from May 2010 to December 2022 at Seoul St. Mary’s Hospital (Seoul, Korea). Five patients with newly diagnosed GD after lobectomy, one patient with distant metastasis of thyroid cancer at initial diagnosis, one patient who underwent the initial operation at a different hospital, two patients with insufficient data, and one patient who was lost to follow-up were excluded from the study. Thus, 122 patients were included in the analysis (**Figure 1**). The mean follow-up duration was 52.8 ± 39.6 months (range, 4.8–144.0 months).

Overweight was defined as a BMI of 25 kg/m² or higher according to the World Health Organization (WHO) and the International Association for the Study of Obesity (IASO) (32). WHO and IASO define obesity as a BMI of 30 or above (33, 34). However, only 7 (5.7%) patients were obese in the present study, according to these criteria (BMI ≥ 30 kg/m²). Moreover, Asian countries have lower cut-off values due to a higher prevalence of obesity-related diseases at lower BMI levels (35). As this study included Korean individuals, the patients were divided by a BMI of 25, which is the standard for overweight defined by WHO and for obesity in Asia (36).

### 2.2 Preoperative management and follow-up assessment

Height and weight were assessed in all patients the day prior to surgery to mitigate potential measurement and temporal biases. BMI was calculated by dividing the weight in kilograms by the square of their height in meters (kg/m2). The duration of GD was defined as the number of years between the date of initial diagnosis and the date of surgery. Disease status was assessed using the serum thyroid function test (TFT), including thyroid stimulating hormone (TSH), triiodothyronine (T3), free thyroxine (T4), and TR-Ab levels before surgery, either as outpatients or after hospital admission. Pathology reports were used to review the final results after surgery.

Patients with GD received treatment based on the 2016 American Thyroid Association (ATA) guidelines for hyperthyroidism (1). Patients with concomitant thyroid cancer were managed according to the 2015 ATA management guidelines for differentiated thyroid cancer (37). After the thyroidectomy, all patients discontinued antithyroid drugs and started taking L-T4 at a daily dosage suitable for their body weight (1.6 μg/kg). Patients with concomitant thyroid cancer were closely monitored every 3–6 months during the first year and then annually thereafter. Thyroid ultrasonography was conducted annually for patients with cancer.

### 2.3 Primary endpoint

The primary endpoint was the rate of overweight in GD patients with and without concomitant thyroid cancer.

### 2.4 Statistical analysis

Continuous variables were reported as means with standard deviations, while categorical variables were presented as numbers with percentages. Continuous variables were compared with Student’s t-tests and Mann-Whitney test, and categorical characteristics were compared using Pearson’s chi-square tests or Fisher’s exact tests. Univariate Cox regression analyses were conducted to determine the biomarkers for postoperative hypoparathyroidism and malignancy in patients with GD. Statistically significant variables were included in the multivariate Cox proportional hazard model. Odds ratios (ORs) with 95% confidence intervals (CIs) were calculated. Statistical significance was defined as p-values < 0.05. The Statistical Package for the Social Sciences (version 24.0; IBM Corp., Armonk, NY, USA) was used for all statistical analyses.

## 3 Results

### 3.1 Baseline clinicopathological characteristics of the study population

**Table 1** presents the clinicopathological characteristics of the 122 patients in the study. The average age was 45.7 years (range, 15–77), and the average BMI was 23.4 kg/m2 (range, 17.2–37.0). 35 patients (28.7%) were classified as overweight. The mean disease duration was 5.9 years, and the mean gland weight was 105.6 grams (range, 7.6–471.4). Most patients (110 patients, 90.2%) underwent total or near-total thyroidectomy; 11 (9.0%) patients underwent lobectomy, and one patient (0.8%) underwent total thyroidectomy with modified radical neck dissection (mRND). The 11 patients who underwent lobectomy exhibited proper regulation of thyroid function prior to surgery, and preoperative diagnosis confirmed the existence of unifocal cancer or follicular neoplasm with a size smaller than 2cm (range, 0.3-1.8). The pathology was benign in 79 (64.8%) patients, while 43 (35.2%) patients exhibited malignant pathology. The preoperative TFT showed a mean TSH level of 1.7 ± 7.8 mIU/L (range, 0.0–77.9), a mean T3 level of 1.7 ± 0.7 ng/mL (range, 0.5–5.1), a mean free T4 level of 1.4 ± 0.7 ng/dL (range, 0.3–4.1), and a mean TR-Ab level of 26.4 ± 35.7 IU/L (range, 0.3–292.8). Forty-four patients (36.1%) underwent surgery due to refractory disease or medication complications, 31 (25.4%) patients underwent surgery due to huge goiters with compressive symptoms, 10 patients (8.2%) underwent surgery due to ophthalmopathies, and 37 (30.3%) patients underwent surgery due to cancer or follicular neoplasm diagnoses before surgery. Postoperative complications were described in **Supplementary Table 1**. Unilateral vocal cord palsy (VCP) occurred in 3 (2.5%) patients, and no bilateral VCP occurred. Hypoparathyroidism was transient in 48 (39.3%) patients and permanent in 3 (2.5%) patients. No cases of hematoma or thyroid storm occurred.

**Table 1:** Baseline clinicopathological characteristics of the study population.

Total 122 patients	
Characteristics	
**Age (years)**	45.7 ± 15.0 (range, 15 - 77)
**Male: Female**	1: 5.8
**Male**	18 (14.8%)
**Female**	104 (85.2%)
**BMI (kg/m^2^)**	23.4 ± 3.8 (range, 17.2 - 37.0)
**Overweight**	35 (28.7%)
**Duration of GD (years)**	5.9 ± 6.3 (range, 0.1 -30.0)
Extent of surgery	
Lobectomy	11 (9.0%)
Total/Near-total	110 (90.2%)
TT with mRND	1 (0.8%)
Gland weight (gm)	105.6 ± 88.0 (range, 7.6 – 471.4)
Pathologic results	
Benign	79 (64.8%)
Malignant	43 (35.2%)
Preoperative management	
None	6 (4.9%)
ATD	9 (7.4%)
KI	9 (7.4%)
ATD + KI	47 (38.5%)
ATD + BB + KI	51 (41.8%)
Preoperative TFT	
TSH (mIU/L)	1.7 ± 7.8 (range, 0.0 - 77.9)
T3 (ng/mL)	1.7 ± 0.7 (range, 0.5 - 5.1)
Free T4 (ng/dL)	1.4 ± 0.7 (range, 0.3 - 4.1)
TR-Ab (IU/L)	26.4 ± 35.7 (range, 0.3 - 292.8)
Cause of surgery	
Refractory, complication to medication	44 (36.1%)
Huge goiter (compressive sx)	31 (25.4%)
Ophthalmopathy	10 (8.2%)
Preoperative dx of cancer or thyroid nodule	37 (30.3%)

Data are expressed as the patient’s number (%) or the mean ± standard deviation. A statistically significant difference was defined as p < 0.05. Abbreviations: BMI, body mass index; GD, Graves’ disease; TT, total thyroidectomy; mRND, modified radical neck dissection; LN, lymph node; ATD, antithyroid drugs; KI, potassium iodide; BB, beta blocker; TFT, thyroid function test; TSH, thyroid stimulating hormone; TR-Ab, TSH receptor antibody; sx, symptom; dx, diagnosis.

### 3.2 Clinicopathological characteristics of thyroid cancer in patients with Graves’ disease

**Table 2** shows the clinicopathological characteristics of the 43 patients diagnosed with thyroid cancer. 42 (97.7%) patients were diagnosed with PTC, while 1 (2.3%) patient had minimally invasive Hürthle cell carcinoma. Thirty-four (79.1%) patients were preoperatively diagnosed with papillary thyroid cancer (PTC) or Hürthle cell neoplasm, while cancers were discovered incidentally in 9 (20.9%) patients. Ten (23.3%) patients underwent lobectomy, 32 (74.4%) patients underwent total or near-total thyroidectomy, and one (2.3%) patient underwent total thyroidectomy with mRND. The most prevalent subtype of PTC was the classic type, accounting for 81.0% of PTC cases. Follicular, tall cell, and oncocytic variants comprised 7.1%, 4.8%, and 7.1% of PTC cases, respectively. The average tumor size was 0.9 cm (range, 0.1–3.4 cm). Multifocalities were observed in 19 (44.2%) patients and bilaterality was observed in 11 (25.6%) patients. Lymphatic invasion, vascular invasion, and perineural invasion were observed in 12 (27.9%), 1 (2.3%), and 2 (4.7%) patients, respectively.

**Table 2:** Clinicopathological characteristics of thyroid cancer in Graves’ disease.

Cancer patients (n= 43)	n (%)
Incidentally discovered cancer	9 (20.9%)
Preoperative diagnosis of nodule	34 (79.1%)
PTC/HN	33(76.7%)/1(2.3%)
Cancer type	
PTC	42 (97.7%)
HCC	1 (2.3%)
Extent of surgery	
Lobectomy	10 (23.3%)
Total/Near-total	32 (74.4%)
TT with mRND	1 (2.3%)
PTC subtype	
Classic	34/42 (81.0%)
Follicular	3/42 (7.1%)
Tall cell	2/42 (4.8%)
Oncocytic	3/42 (7.1%)
**Tumor size (cm)**	0.9 ± 0.7 (range, 0.1 - 3.4)
Extrathyroidal extension	
Minimal	16 (37.2%)
Gross	2 (4.7%)
**Multifocality**	19 (44.2%)
**Bilaterality**	11 (25.6%)
**Lymphatic invasion**	12 (27.9%)
**Vascular invasion**	1 (2.3%)
**Perinueral invasion**	2 (4.7%)
**BRAF^V600E^ positivity**	26/31 (83.9%)
**TERT positivity**	1/17 (5.9%)
T stage	
T1/T2/T3b	38 (88.4%)/3 (7.0%)/2 (4.7%)
N stage	
N0,Nx/N1a/N1b	33 (76.8%)/9 (20.9%)/1 (2.3%)
TNM stage	
Stage I/II	40 (93.0%)/3 (7.0%)

Data are expressed as the patient’s number (%) or the mean ± standard deviation. A statistically significant difference was defined as p < 0.05. Abbreviations: PTC, papillary thyroid cancer; HTC, Hürthle cell thyroid cancer; HN, Hürthle cell neoplasm; mRND; modified radical neck dissection.

As shown in **Table 3**, the 34 patients who were preoperatively diagnosed with cancers were compared with the 9 patients with incidentally discovered cancers after surgery. No differences in BMI were detected between the two groups (23.3 ± 3.7 vs. 24.4 ± 4.1; *p* = 0.450). Gland weight was significantly lighter in patients with preoperatively diagnosed cancers compared with gland weights in the incidentally discovered group (35.3 ± 40.1 vs. 119.2 ± 62.9; *p* < 0.001). TR-Ab levels were significantly lower in the preoperatively diagnosed group compared with the levels in the incidentally discovered group (5.5 ± 5.3 vs. 31.4 ± 28.9; *p* = 0.005). Tumor size was significantly larger in the preoperatively diagnosed group compared with the size in the incidentally discovered group (1.0 ± 0.7 vs. 0.4 ± 0.2, *p* = 0.001). The causes of surgery were also significantly different between the two groups (*p* < 0.001). In the incidentally discovered cancer group, 66.7% of the patients underwent surgery due to refractory disease or medication complications, 22.2% due to large goiters, and 11.1% due to nodules detected on preoperative ultrasound. In contrast, all surgeries were performed due to the preoperative detection of cancer in the group with preoperative diagnosis.

**Table 3:** Comparison of thyroid cancers in Graves’ disease with or without preoperative pathologic diagnosis.

Characteristics	Pre-op dx (-) (n=9)	Pre-op dx (+) (n=34)	*p*-value
**Age (years)**	49.1 ± 19.0 (range, 30 - 75)	47.7 ± 12.0 (range, 15 -67)	0.880 †
**BMI (kg/m^2^)**	23.3 ± 3.7 (range, 18.7 - 29.4)	24.4 ± 4.1 (range, 17.2 -33.2)	0.450 ‡
**Duration of GD (years)**	5.8 ± 3.6 (range, 1 - 11)	3.4 ± 4.0 (range, 0.1 - 15)	0.021 †
**Gland weight (g)**	119.2 ± 62.9 (range, 31.2 - 219.9)	35.3 ± 40.1 (range, 7.6 -236.8)	<0.001 †
Preoperative TFT			
TSH (mIU/L)	0.2 ± 0.2 (range, 0.01 - 0.69)	3.4 ± 13.3 (range, 0.01 - 77.9)	0.450 †
T3 (ng/mL)	1.6 ± 0.7 (range, 0.7 - 2.7)	1.3 ± 0.5 (range, 0.5 - 3.5)	0.275 †
Free T4 (ng/dL)	1.1 ± 0.7 (range, 0.3 - 2.2)	1.4 ± 0.5 (range, 0.4 - 2.5)	0.213 ‡
TR-Ab (IU/L)	31.4 ± 28.9 (range, 2.0 - 88.0)	5.5 ± 5.3 (range, 0.3 - 21.0)	0.005 †
**Tumor size (cm)**	0.4 ± 0.2 (range, 0.1 - 0.7)	1.0 ± 0.7 (range, 0.3 - 3.4)	0.001 †
**Cause of surgery**			<0.001
Refractory or complication to medication	6 (66.7%)	0 (0%)	
Huge goiter	2 (22.2%)	0 (0%)	
Pre-op nodule on US	1 (11.1%)	34 (100%)	

Data are expressed as the patient’s number (%) or the mean ± standard deviation. †:significance level by the Mann-Whitney test, ‡:significance level by the Student’s T-test. A statistically significant difference was defined as p < 0.05. Abbreviations: pre-op dx, preoperative diagnosis of cancer; BMI, body mass index; GD, Graves’ disease; TSH, thyroid stimulating hormone; TR-Ab, TSH receptor antibody; US, ultrasound.

### 3.3 Comparison of Graves’ disease subgroups with or without thyroid cancer

Patients with GD with or without thyroid cancer were compared, as shown in **Table 4**. Patients with GD and thyroid cancer were significantly more overweight (BMI ≥ 25 kg/m2) than patients with GD without thyroid cancer (44.2% vs. 20.3%; *p* = 0.005). The duration of GD was longer in patients without cancer than the duration in patients with cancer (6.9 ± 7.1 vs. 3.9 ± 4.0 years; *p* = 0.003). Gland weights were significantly heavier in patients without cancer compared with patients with cancer (134.7 ± 88.9 vs. 52.9 ± 56.6 g; *p* < 0.001). Preoperative TR-Ab was significantly higher in patients without cancer compared with TR-Ab levels in patients with cancer (34.9 ± 40.1 vs. 10.9 ± 17.2 IU/L; *p* < 0.001).

**Table 4:** Comparison between sub-groups of Graves’ disease with or without thyroid cancer.

Characteristics	without Cancer (n = 79)	with Cancer (n = 43)	*p* - value
**Age**	44.4 ± 15.7 (range, 18 -77)	48.0 ± 13.5 (range, 15 -75)	0.210
**Gender**			0.854
Female	67 (84.8%)	37 (86.0%)	
Male	12 (15.2%)	6 (14.0%)	
**Overweight** (BMI > 25 kg/m^2^)	16 (20.3%)	19 (44.2%)	0.005
**Duration of GD** (years)	6.9 ± 7.1 (range, 0.1 - 30.0)	3.9 ± 4.0 (range, 0.1 - 15.0)	0.003
**Gland weight** (g)	134.7 ± 88.9 (range, 20.8 - 471.4)	52.9 ± 56.6 (range, 7.6 - 236.8)	<0.001
**TR-Ab**	34.9 ± 40.1 (range, 0.3 - 292.8)	10.9 ± 17.2 (range, 0.3 - 88.0)	<0.001

Data are expressed as the patient’s number (%) or the mean ± standard deviation. A statistically significant difference was defined as p < 0.05. Abbreviations: BMI, body mass index; GD, Graves’ disease; TR-Ab, TSH receptor antibody.

### 3.4 Univariate and multivariate analyses of biomarkers for malignancy in patients with Graves’ disease

Univariate analysis revealed that being overweight, the duration of GD, gland weight, and preoperative TR-Ab were significant biomarkers for malignancy in patients with GD (**Table 5**). In the multivariate analysis, being overweight, lighter gland weight, and lower postoperative TR-Ab levels were confirmed as biomarkers for malignancy. Being overweight emerged as the most significant biomarker for malignancy (OR, 3.108; 95% CI, 1.196–8.831; *p* = 0.021).

**Table 5:** Univariate and multivariate analyses of biomarkers for malignancy in patients with Graves’ disease.

	Univariate analysis		Multivariate analysis	
	OR (95% CI)	*p*-value	OR (95% CI)	*p*-value
Age	1.016 (0.991 ;- 1.042)	0.209		
Female	1.104 (0.383 - 3.185)	0.854		
Overweight (BMI > 25 kg/m^2^)	3.117 (1.381 - 7.038)	0.006	3.108 (1.196 - 8.831)	0.021
Duration of GD	0.908 (0.840 - 0.982)	0.016	0.968 (0.882 - 1.063)	0.501
Gland weight (g)	0.979 (0.970 - 0.988)	<0.001	0.984 (0.975 - 0.994)	0.002
TR-Ab	0.939 (0.911 - 0.968)	<0.001	0.964 (0.933 - 0.997)	0.033

A statistically significant difference was defined as p < 0.05. Abbreviations: OR, odds ratio; CI, confidence interval; BMI, body mass index; GD, Graves’ disease; TR-Ab, TSH receptor antibody.

## 4 Discussion

The present study aimed to investigate the biomarkers for concomitant thyroid cancer in patients with GD and identify the effects of being overweight on cancer risk. Patients with GD and concomitant thyroid cancer were more likely to be overweight compared to patients with GD without cancer. In addition, overweight patients had a significantly increased risk of developing thyroid cancer compared to non-overweight patients.

In GD, TR-Ab stimulates the TSH receptor, leading to increased production and release of thyroid hormones. Excessive thyroid hormone affects entire body tissues, including thermogenesis and metabolic rate. GD symptoms vary by hyperthyroidism severity and duration (1, 2, 31).

The reported incidence of concomitant thyroid cancer in GD ranges from 1% to 22% (8–11, 38, 39). Since this study included GD patients who meet the surgical indications, the cohort demonstrated a higher prevalence of thyroid cancer compared to the general GD population. The frequency of cancer in patients with GD is consistent with the frequency in the general population. All types of thyroid cancer can occur in GD patients; PTC is the most common cancer followed by FTC (8, 40). While surgery is not the primary treatment for GD, surgical intervention may be performed in cases that meet specific surgical indications (1, 2). According to the 2016 ATA guidelines for hyperthyroidism, near-total or total thyroidectomy is recommended for surgical intervention of GD (1). However, 11 patients underwent lobectomy in our study; these patients maintained a euthyroid state with preoperatively detected nodules, and the decision to perform lobectomy was made based on the individual preferences of the patients and the multidisciplinary medical team. GD did not recur in any of the 11 patients who underwent lobectomies. 

Numerous studies have demonstrated that thyroid cancer is more aggressive in obese and overweight patients, irrespective of the coexistence of GD (26–28, 41, 42). In a case-control study, Marcello et al. showed that being overweight (BMI ≥ 25 kg/m2) is associated with an increased risk of thyroid cancer (OR, 3.787; 95% CI, 1.110–6.814, p < 0.001) (27). GD is a hypermetabolic disease, which usually causes weight loss, and obesity is not common in patients with GD (31). Weight gain is a useful indicator for evaluating initial treatment success for hyperthyroidism, but weight loss should be considered differently in obese patients. Hoogwerf et al. reported that despite greater weight loss at the time of the initial diagnosis of GD, obese patients were still morbidly obese and had higher thyroid function values compared to non-obese patients (43). The diagnosis of hyperthyroidism may be delayed in these patients as weight loss is often perceived as a positive outcome. The results of our study agree with earlier studies and are supported by an OR of 3.108, which is similar to the OR of 3.787 reported in the Marcello study (27).

The mean tumor size in this study was 0.9 cm, which was similar to previous studies concerning thyroid cancer in patients with GD. In a study by Hales et al., the average size of thyroid cancer in patients with GD was 0.91 cm, which was significantly smaller than the average size in the euthyroid group (0.91 vs. 2.33 cm) (44). However, previous studies demonstrated a more aggressive thyroid cancer phenotype in patients with GD (9, 45). In addition, Marongju et al. revealed a higher degree of aggressiveness in some patients with microcarcinoma and GD compared to controls, even when tumor characteristics were favorable, which conflicts with other studies (45). The presence of both thyroid cancer and GD is a surgical indication, regardless of the size of the cancer. Thus, microcarcinoma in GD should not be overlooked.

Lower preoperative TR-Ab were biomarkers for malignancy in patients with GD in this study. TR-Ab, which promotes hyperthyroidism by inducing the production and release of thyroid hormones, is a diagnostic biomarker for GD (13, 20). Several studies have explored the link between TR-Ab and concurrent thyroid cancer in patients with GD and showed that TR-Ab can potentially trigger thyroid cancer by continuously stimulating thyroid cells (20, 46). However, other studies did not detect an association between TR-Ab and concomitant thyroid cancer in patients with GD, which is consistent with our findings (16, 40, 47). Yano et al. demonstrated that elevated TR-Ab was significantly associated with smaller tumor size in patients with GD and had no significant impact on multifocality or lymph node metastasis (40). Similarly, Kim et al. concluded that the behavior of thyroid cancer is not affected by TR-Ab (16). We attributed these results to the fact that patients with GD and cancer may undergo surgery due to the detection of nodules that were relatively well-controlled with medication for a long time. On the other hand, in the GD without cancer group, surgery is often performed due to uncontrolled hyperthyroidism despite medication, and TR-Ab levels may be higher. Future research should investigate the association between TR-Ab levels and thyroid cancer risk in larger studies to clarify the contradictory findings in previous studies.

The lighter gland weight was a biomarker for the concomitant thyroid cancer; however, measuring the gland weight before surgery is not feasible in clinical practice. Nonetheless, ultrasound can estimate thyroid volume preoperatively using the ellipsoidal formula: Volume = (π/6) × Length × Width × Depth. The overall thyroid volume can be derived by adding together the volume calculations for both lobes (48). Future studies will focus on applying this method clinically and investigating the link between preoperative thyroid dimensions and the prevalence of concomitant thyroid cancer.

This study’s strengths include long follow-up duration with more than 100 patients, providing robust results. Additionally, the study included various demographic and clinical factors, providing a comprehensive evaluation of thyroid cancer biomarkers in patients with GD. Of note, this study focused on the effect of being overweight in patients with GD, rather than the general population. However, the relationship between GD, thyroid cancer, and overweight is complex and may involve a variety of factors, including genetics, hormonal imbalances, and lifestyle factors.

This study has several limitations. First, its retrospective design and relatively small sample size may have introduced selection and information bias. Second, the study was conducted in the Korean population, limiting generalizability to other populations. Lastly, BRAF and TERT assessments were conducted in a limited cohort, insufficient to represent the entire study population, and there is a paucity of data on the molecular characteristics and genetic information for thyroid cancer. Further research should investigate the effects of being overweight on thyroid cancer risk in a diverse population of patients with GD to determine whether the results are generalizable. In addition, more investigations into the long-term postoperative outcomes of patients with GD with and without concomitant thyroid cancer may provide a more comprehensive evaluation of surgical outcomes.

## 5 Conclusions

Overweight individuals with GD have a higher risk of developing concomitant thyroid cancer. This highlights the importance of thorough screening and comprehensive evaluations specifically tailored to overweight GD patients to detect and prevent thyroid cancer. Further research is needed to elucidate the underlying mechanisms and the effects of being overweight on thyroid cancer risk in GD patients in the general population.

## Data availability statement

The raw data supporting the conclusions of this article will be made available by the authors, without undue reservation.

## Ethics statement

The studies involving humans were approved by Institutional Review Board of Seoul St. Mary’s Hospital, The Catholic University of Korea (IRB No: KC23RISI0054 and date of approval: 2023.04.21). The studies were conducted in accordance with the local legislation and institutional requirements. The ethics committee/institutional review board waived the requirement of written informed consent for participation from the participants or the participants’ legal guardians/next of kin because due to the retrospective nature of this study.

## Author contributions

JP: Writing – review & editing, Writing – original draft, Visualization, Validation, Investigation, Formal analysis, Data curation, Conceptualization. SA: Writing – review & editing, Software, Data curation. JB: Writing – review & editing, Supervision, Software, Resources, Methodology. JK: Writing – review & editing, Supervision, Resources, Methodology. KK: Writing – review & editing, Writing – original draft, Visualization, Validation, Software, Resources, Project administration, Methodology, Investigation, Formal analysis, Data curation, Conceptualization.
