# Supplementary material for: Overweight as a biomarker for concomitant thyroid cancer in patients with Graves’ disease
Source: Front Endocrinol (Lausanne). 2024 Apr 22;15:1382124. doi: 10.3389/fendo.2024.1382124 (PMC11070936; doi:10.3389/fendo.2024.1382124)
Supplement: Supplementary file 1 [file Table_1.docx]

Supplementary Material

# Supplementary Table

## Supplementary Table 1.

**Supplementary Table 1**. Complications after thyroidectomy in Graves’ disease.

| Complication | n (%) |
| --- | --- |
| **Vocal cord palsy** |  |
| Unilateral | 3 (2.5%) |
| Bilateral | 0 (0%) |
| **Hypoparathyroidism** |  |
| Transient | 48 (39.3%) |
| Permanent | 3 (2.5%) |
| **Hematoma** | 0 (0%) |
| **Thyroid storm** | 0 (0%) |

Data are expressed as the patient’s number (%).

## Supplementary Table 2. Significant variables in comparison of thyroid cancers in Graves’ disease with or without preoperative pathologic diagnosis with statistical power.

| Characteristics | Pre-op dx (-) (n=9) | Pre-op dx (+) (n=34) | *p*-value | Power (%) |
| --- | --- | --- | --- | --- |
| **Gland weight (g)** | 119.2 ± 62.9  (range, 31.2 - 219.9) | 35.3 ± 40.1  (range, 7.6 -236.8) | <0.001 | 31.5 |
| **TR-Ab (IU/L)** | 31.4 ± 28.9  (range, 2.0 - 88.0) | 5.5 ± 5.3  (range, 0.3 - 21.0) | 0.005 | 20.3 |
| **Tumor size (cm)** | 0.4 ± 0.2  (range, 0.1 - 0.7) | 1.0 ± 0.7  (range, 0.3 - 3.4) | 0.001 | 24.4 |

Data are expressed as the patient’s number (%) or the mean ± standard deviation. A statistically significant difference was defined as *p* < 0.05. Abbreviations: pre-op dx, preoperative diagnosis of cancer; TR-Ab, TSH receptor antibody.

## Supplementary Table 3. Significant variables in comparison between sub-groups of Graves’ disease with or without thyroid cancer with statistical power.

| Characteristics | without Cancer (n = 79) | with Cancer  (n = 43) | *p* - value | Power (%) |
| --- | --- | --- | --- | --- |
| **Overweight**  (BMI > 25 kg/m^2^) | 16 (20.3%) | 19 (44.2%) | 0.005 | 99.84 |
| **Gland weight** (g) | 134.7 ± 88.9  (range, 20.8 - 471.4) | 52.9 ± 56.6  (range, 7.6 - 236.8) | <0.001 | 97.94 |
| **TR-Ab** | 34.9 ± 40.1  (range, 0.3 - 292.8) | 10.9 ± 17.2  (range, 0.3 - 88.0) | <0.001 | 64.12 |

Data are expressed as the patient’s number (%) or the mean ± standard deviation. A statistically significant difference was defined as *p* < 0.05. Abbreviations: BMI, body mass index; TR-Ab, TSH receptor antibody.
